# Mainstreaming domestic and gender-based violence into sociology and the criminology of violence

**DOI:** 10.1111/1467-954X.12198

**Published:** 2014-12-16

**Authors:** Sylvia Walby, Jude Towers, Brian Francis

**Keywords:** violence, domestic violence, crime, victim-offender relationship, gender, measurement, quantitative methods

## Abstract

Sociological and criminological views of domestic and gender-based violence generally either dismiss it as not worthy of consideration, or focus on specific groups of offenders and victims (male youth gangs, partner violence victims). In this paper, we take a holistic approach to violence, extending the definition from that commonly in use to encompass domestic violence and sexual violence. We operationalize that definition by using data from the latest sweep of the Crime Survey for England and Wales. By so doing, we identify that violence is currently under-measured and ubiquitous; that it is gendered, and that other forms of violence (family violence, acquaintance violence against women) are equally of concern. We argue that violence studies are an important form of activity for sociologists.

## Introduction

Žižek (2009 [2008]) is wrong to argue that sociologists should refrain from examining direct violence on the grounds that this distracts from more important matters. Bourdieu (2000 [1997]2000 [1997]) is wrong to argue that violence involves the complicity of those who undergo it. These men underestimate the importance of violence in the lives of women; the significance of visceral physical force and the harm that it causes. The scholarly neglect of domestic violence and other forms of violence against women has a long heritage. Weber (1948) thought the modern state had a monopoly of legitimate violence in its territory, even at a time when rape and violence in the domestic sphere were not crimes when committed by husbands against wives. Merton (1938) was one of many sociologists and criminologists to locate violence as the product of socio-economic inequalities, studying young disadvantaged men; but allowed their victims to remain largely invisible.

The mainstream neglect is embedded in the construction of public knowledge. Taking England and Wales as an exemplar, the official count of violent crime, police recorded crimes, has no categories in which to capture domestic violence or gender-based violence (with the exception of some sexual offences); thus violent crime against women is routinely made invisible in the public sphere.

Beyond the analysis of inter-personal violence there is work on other forms of violence – that of violence of states, militaries and political movements (Tilly, 2003; Mann, 1986), but this is beyond the scope of this paper, though see Walby (2009, 2013), Ray (2011), Wieviorka (2009) and Malešević (2010).

There has been a growing challenge to the gendered assumption that domestic violence and gender-based violence against women are not important. There is an emerging epistemic community (Haas, 1992), combining the development of new knowledge and practices, involving feminist activists, service providers, academics and public officials. The violence emerges into public view in the form of ‘scandals’, when some famous man is accused of perpetrating gendered violence (eg Jimmy Saville, Julian Assange, Strauss-Kahn), or when a particularly horrendous incident stirs public outrage (eg sex abuse of children by Catholic priests; gang rape in India). But this particular knowledge is not yet sedimented into authoritative academic knowledge, although there are significant attempts to achieve this (eg Walklate, 2004). As Sharp (2006: 3) notes, in her editorial on the launch of the journal *Feminist Criminology*, ‘the majority of criminological research in the top-tier journals still either ignores women or treats gender as a control variable’.

There is an emerging field at the intersection of and in the interstices between sociology, gender studies, criminology, social policy and social statistics that investigates and analyses domestic and gendered violence. This field includes debates as to the extent to which: domestic violence is gendered (Straus and Gelles, 1990; Dobash *et al*., 1992; Archer, 2000); domestic abuse is violent (Stark, 2007); and frequent repetitions of domestic violence can be counted (Johnson, 1995, 2008).

Despite its vibrancy, the field of domestic and gendered violence has developed relatively separately from mainstream disciplines. It has developed its own conferences and journals; its own theories, concepts and forms of measurement; its priority fields of enquiry. This separate development has consequences for the integration of the scholarship and research on domestic and gendered violence into mainstream sociology and criminology that are not always positive. The purpose of this paper is to mainstream the analysis of domestic and gendered violence into sociology. This is not merely a one-way impact, but a process of mutual adaptation of these two complex systems of thought.

By mainstreaming violence, this paper is able to pursue a challenge to orthodox views by investigating the extent to which interpersonal violence is domestic and is gendered, using a form of knowledge widely considered to be authoritative: statistics. It rejects the view that official statistics are inevitably part of the dominant order; arguing that instead they can be used to speak ‘truth to power’. It contributes to the agenda of making visible this violence by addressing and solving some of the dilemmas and complexities of measurement necessary to identify the violence as a robust object for analysis. It draws on analysis of a range of sources of data including, in particular, the Crime Survey for England and Wales (CSEW), formerly the British Crime Survey.

As part of this mainstreaming, there is also a need for some of the particularities of the challenger field of gendered violence to be modified. This includes reconsideration of concepts and measurements so as to be compatible with the mainstream. There is need to explicitly address the less than full overlap of the violence that is variously ‘domestic’, ‘gender-based’ and ‘against women’. This includes consideration of violence that is gendered, but not domestic.

In this way, the paper develops further the critique of current theoretical frameworks in which domestic violence and gender-based violence are marginalized; identifies the scale of domestic and gendered violence as a proportion of all violent crime; demonstrates the implications of different definitions of domestic and gendered violence for substantive findings and analysis; and establishes the significance of domestic and gendered violence to social theory.

## Review

The neglect of gendered inter-personal violence is widespread in contemporary social theory. This occurs in two main ways. First, violence is defined in such a way as to position its harm as if it derives abstractly from systems of inequality, thereby obscuring physical harms and physical violence from men to women, as illustrated by the work of Žižek and Bourdieu. Second, within the analysis of violence within the field of sociological criminology, there is segregation of mainstream accounts of violence that leave gender out of focus from an emerging specialized school of gender-based violence. Although there are attempts to merge the insights from the specialized analysis of gendered violence into mainstream theory in this area (eg Walklate, 2004; Hearn, 2012), they have had limited impact. We identify some of the reasons for this lack of impact in order to suggest how they may be addressed, thereby offering a route to more successful mainstreaming of the analysis of gendered violence into the analysis of inter-personal violence and into social theory.

### Social theory: Žižek and Bourdieu

Žižek (2009 [2008]: 10) argues that the focus on, the ‘fascination’ with, direct violence, or ‘subjective violence’, is a distraction from more important systemic issues. He distinguishes between ‘subjective violence – that violence which is enacted by social agents, evil individuals, disciplined repressive apparatuses, fanatical crowds’ (2009: 9) and ‘the fundamental systemic violence of capitalism … that is no longer attributable to concrete individuals and their “evil” intentions, but is purely “objective”, systematic, anonymous’ (2009: 11). He concludes thus ‘to chastise violence outright, to condemn it as ‘bad’, is an ideological operation par excellence, a mystification which collaborates in rendering invisible the fundamental forms of social violence’ (2009: 174).

Žižek is right to note that the prioritization of different forms of violence is not a simple given but is socially variable and influenced by media and other social practices. But his examples of victims, starting with elites who suffer from revolutionary violence, suggest that he is unaware of the actual distribution of victims of violence in unequal societies. His prioritization of the ‘objective’ wider system is insensitive to these actual patterns in inter-personal violence, in which women and other minoritized groups are further harmed. His ostensibly radical argument that it is important to focus on ‘the system’ not its immediate victims merely rehearses the same old gender-blind practices of the traditional left.

Bourdieu (2000), like Žižek, also seeks to displace physical violence from the centre of attention of analysis, but by a different theoretical manoeuvre. Bourdieu broadens the concept of violence so that it overlaps with and is indistinguishable from that of power. This might initially appear to expand the scope of analysis, but in fact it merely erodes the specificity and potential for distinctive explanatory power of the concept of violence, since if violence is merely symbolic power, then it is no more than other forms of symbolic power. Indeed, Bourdieu goes further and suggests that victims of violence are complicit in this violence because they have learned their position within the practices of power and these are deeply sedimented in their habitus and embedded in their bodies. Bourdieu (2000: 169–170) writes of

the inscription of a relation of domination into the body … The practical recognition through which the dominated, often unwittingly, contribute to their own domination by tacitly accepting, in advance, the limits imposed on them … submitting, however reluctantly, to the dominant judgement, sometimes in internal conflict and ‘self-division’, the subterranean complicity that a body slipping away from consciousness and will maintains with the violence of the censures inherent in the social structures.

Symbolic violence is the coercion which is set up only through the consent that the dominated cannot fail to give to the dominator. … The effect of symbolic domination (sexual, ethnic, cultural, linguistic, etc) is exerted not in the pure logic of knowing consciousness but in the obscurity of the dispositions of habitus, in which are embedded the schemes of perception and appreciation … below the level of the decisions of the conscious mind and the controls of the will.

In consequence, Bourdieu cannot theorize violence as separate from other forms of power; he cannot identify its specific character, rhythms and modalities. The concept of ‘symbolic violence’ refuses the specificity of the visceral, the particularity of the power that comes from control over physical pain rather than over economic resources. In so doing he is rejecting a conceptual distinction that is relevant to the analysis of the modalities of power. Even more odd is his claim that those who suffer symbolic violence collaborate with and are complicit with their oppressor rather than resist and fight back. Such a position is rejected by Fanon (19901990 [1967]), who details the ever-present awareness of the oppressed of the risk of violence from the hated colonial oppressor (see also von Holdt, 2013). Bourdieu's position is the opposite of that of Gramsci (1971), for whom coercion made visible the relations of domination that could in less contentious times be disguised by consent.

These critical social theorists, Žižek and Bourdieu, not only marginalize gendered violence, but also inter-personal violence more generally. Žižek argues that the abstract systemic ‘violence’ of capitalism is more important than the concrete examples of direct violence that are picked up in the media, while Bourdieu refuses the specificity of violence distinct from other forms of symbolic power. If ‘theory’ is so problematic, will matters improve when looking at the empirical social science closest to violence, criminology?

### Criminology

Most of the empirical analysis of inter-personal violence in the social sciences now takes place in the field of criminology, though this overlaps with sociology and other disciplines, and within a relatively segregated field of ‘violence against women’. Much, though not all, criminology subsumes violent crime into a general crime category, except where the analyses are more specific to crime type. Criminology is internally diverse, being subdivided into several competing and overlapping schools of thought (Maguire *et al*., 2012; McLaughlin *et al*., 2003; Newburn, 2013); hence, the generalizations that are made below come with warnings about the need for caution and caveats. Nevertheless, it is not unreasonable to identify the following schools of thought, theories and paradigms: structural strain theory (Merton, 1938), many times reworked (Agnew, 1992, 1999), which branches into the Chicago school of social disorganization (Shaw and McKay, 1942), the inequality/relative deprivation school (Young, 1999), and related approaches to macro level sources of variation (Pratt and Cullen, 2005); and rational choice theory originating in economics (Becker, 1968), which informs the development of the routine activity paradigm (Cohen and Felson, 1979), the self-control deficit paradigm (Gottfredson and Hirschi, 1990) and the criminal career paradigm (Piquero *et al*., 2003) as well as economic criminology (Cook *et al*., 2013). Gender is remarkable by its absence from these schools of thought, appearing instead as a separate field. In textbooks and overviews of criminology gender will often appear as a separate chapter that is ill-integrated into the rest of the text (see, for example, Miller and Mullins, 2008).

In the classic sociological account by Merton, the cause of crime lay in the ‘malintegration’, or structural strain between ‘culturally defined aspirations’ and ‘socially structured means’ of obtaining them (Merton, 1938: 674).

The fact that actual advance toward desired success symbols through conventional channels is, despite our persisting open-class ideology, relatively rare and difficult with those handicapped by little formal education and few economic resources... On the one hand, they are asked to orient their conduct toward the prospect of accumulating wealth and on the other they are largely denied effective opportunities to do so institutionally. The consequences of such structural inconsistency are psycho-pathological personality, and/or antisocial conduct, and/or revolutionary activities. (Merton, 1938: 679)

Crime is thus one of the outcomes of structural strain between culturally defined aspirations and class-based inequalities in the means to fulfil them. Merton situates the causality of crime at the level of social structure, in the tensions generated by socially structured inequalities. Yet, despite gender relations being deeply structured by inequalities, gender is omitted from this foundational account of crime.

More recent work develops the analysis of the implications of structural inequality for crime, including violent crime, through the route of both inequality and relative deprivation and also that of poverty and social disorganization. The conceptual focus on relative deprivation lends itself to synergy with political economy and left realism (Young, 1999). For example, there are studies that link cross-national rates of homicide with economic inequality and poverty (Cole and Gramajo, 2009; Pridemore, 2008; van Wilsem, 2004), including Fajnzylber *et al*. (2002) who find not only a statistically significant correlation of the link between economic inequality and homicide in a cross-national data set over time, but also evidence that inequality is the cause of changes in its rate. The focus on social disorganization, developed by the ‘Chicago school’ (Shaw and McKay, 1942), is focused on the social disorganization that can be the consequence of poverty, but which is mediated by the different ways civil society and the social relations within neighbourhoods are constituted (Sampson *et al*., 2002). There is a very large body of empirical evidence linking economic inequality, variously operationalized as poverty, income inequality, unemployment, poverty and disadvantage, with crime including violent crime, at both individual and macro levels. A very strong correlation is found in several major reviews, including a meta-review of 63 studies by Chiricos (1987), of 34 studies by Hsieh and Pugh (1993), and the meta-analysis of over 200 studies by Pratt and Cullen (2005). The finding across these myriad of studies, methodologies and perspectives is that crime, including violent crime and homicide, is perpetrated by the disadvantaged. Yet, there is little discussion of gender, despite the significance of gender inequalities.

In the work informed by the more individualistic traditions of psychology and economics, such as rational choice theory (eg Becker, 1968), the link between disadvantage and violent crime re-emerges, but is articulated through different causal pathways. In rational choice theory, crime is committed when the benefits of crime outweigh its costs in the sanctions that might be brought to bear on the perpetrator (Becker, 1968) by the state or civil society. In studies within the self-control deficit paradigm (Gottfredson and Hirschi, 1990), the correlation that is discovered between harsh and poor upbringing and later criminality is explained by variations in self-control: poor upbringing in early childhood causes poor self-control, which causes crime because such individuals find it harder to resist temptation. This paradigm has become one of the most important in contemporary criminology, with a meta-analysis of 21 studies finding that self-control was a strong predictor of crime (Pratt and Cullen, 2000). This paradigm meshes with the criminal career paradigm (Piquero *et al*., 2003), in which poor upbringing results in a criminal career, and the worse the upbringing the earlier the onset and later the desistance from crime. Although these methodologically individualist approaches might appear to float free from social structurally generated inequalities and disadvantages, in fact this is not entirely the case, since they are brought in by the back door of the social conditions that shape the family circumstances that lead to the development of individuals with low self-control, while the existence of non-linear effects (Mears *et al*., 2013) suggests the relevance of mediating social institutions. Again women are largely invisible, though with a few exceptions.

Additionally, gang theories of violence have been proposed. For example, there may exist a normative acceptance of violence in some societal groups (Wolfgang and Ferracuti, 1967). Such values are transmitted within the cultural group, and individuals can also be born into such a subculture. The theory has proved controversial as it has become associated with ethnic or black violence, but Wolfgang and Ferracuti also identified social class as a relevant societal group.

There are some specific criminological theories of individualized violence that are not general theories of crime. Such authors turn to psychopathological explanations of violent behaviour such as conduct disorder or sociopathic tendencies. Moffit (1993) identifies brain injury and abnormal brain activity as associated with life-course persistent offending – a small percentage of offenders who are most likely to be involved in violence. However, she goes on to state that social factors such as poverty and childhood upbringing will interact with these biological factors – children can overcome psychological deficit through successful upbringing.

Most criminology, therefore, suggests that violence and crime are generated by the disadvantaged, in one way or another, with many empirical studies consistent with this paradigm. Women appear, if at all, as an absence, as the mother who failed to socialize her children into appropriate levels of self-control. Women as the victims of male violence are rarely visible in mainstream criminology, being treated as a separate field. This is beginning to be seen as a problem. For example, the meta-analysis of studies on self control and victimology notes the absence of studies on intimate partner violence, violence against women, family violence, and child abuse and concluded ‘self-control theory cannot assume the flavour of generality for which it was originally intended’ until these studies are included (Pratt *et al*., 2014: 90).

### Gender-based violence against women

A specialist sub-field of gender-based violence against women has developed over the last 30 years. It has developed by identifying, naming, describing, documenting, counting, and analysing an increasing range of forms of violence. As part of a wider epistemic community, alongside activists, service providers and policymakers, this academic field has helped to bring the issues of gender-based violence against women into public view, established a field of political activism, steady policy development, and increasingly a field of social scientific enquiry with journals and conferences. Most of the field is self-contained, with only a limited literature that simultaneously addresses the specialist and general analytic fields. There are several theoretical and methodological divergences between the mainstream and gendered fields that are conducive to their continued segregation: first, in theoretical assumptions; second, in definition of violence; third in the significance of the relationship between offender and victim; fourth in whether the extent of the violence concerns the number of violent events or the number of victimized people.

Contrary to much criminology, most of the analysis of gender-based violence treats this violence to be primarily from the advantaged (largely men) and directed primarily towards the disadvantaged (largely women). Gender-based violence against women is commonly seen as both a consequence and a cause of gender inequality (Dobash and Dobash, 1979; Kelly, 1988). The direction of violence, from advantaged to disadvantaged, is different from much mainstream criminology where such directionality of violence is not part of the dominant paradigm. This theoretical divergence hinders the integration of the two fields. An example of such work in the field of domestic violence is that of Kalmuss and Straus (1982) that found the greater were the objective intra-household gender inequalities, the more likely that conflict would lead to violence. Objective dependency was considered to occur when the wife was not employed, when her husband earned more than 75 per cent of the couple's income, and when there were the further constraint of children aged five or younger at home. However, there are both exceptions to this generalization and also multiple ways in which ‘gender inequality’ is interpreted and operationalized. For example, there is a minority tradition in the field, which argues that domestic violence is perpetrated by women as much as by men; which rejects the claim of the link between gender disadvantage and domestic violence, suggesting instead that women can be as violent as men and that family dysfunction is the main cause of the violence (Straus, 1979). While there are claims that studies support this view (Archer, 2000), the weight of the evidence in the field does not support this view of gender symmetry of the perpetrators of the violence (Dobash *et al*., 1992; Walby and Allen, 2004). But the issue as to the nature of relative and absolute gender inequalities and ascertaining which of diverse potential causal pathways between inequality and violence are the most important is not yet fully resolved. Vieraitis *et al*. (2007) find that variations in women's absolute status correlate with their risk of being victims of homicide, but not their relative position, in a cross-sectional analysis of US counties in 2000. Pridemore and Freilich (2005) find a positive relationship between gender income equality and women being victims of homicide, in a cross-sectional study in the US, which they interpret as caused by a backlash from conservative men to reductions in gender inequality; though they note caveats and limitations in that there was no correlation with the strength of a masculine subculture. Further, there are complex interactions between different inequalities, including gender and ethnicity as well as those of gender and socio-economic position (Crenshaw, 1991; Burgess-Proctor, 2006; Bernard, 2013). The divergence in the assumptions about the disadvantaged position of perpetrator and victim in the mainstream and specialist field presents challenges to the integration of gender-specific and general theories of violent crime.

The second divergence between the mainstream and gender fields stems from tension between a definition of violence that focuses on criminalized physical actions and another that extends this to encompass many other forms of power, which is reflected in different measures of the nature and severity of the acts in mainstream crime analysis and in the gender violence field (Walby, 2013). This debate has parallels in fields of research on violence (de Haan, 2009). The mainstream crime field deploys a set of specific categories built up over years of development of nationally based criminal law, while the gender violence field, through developments in international policy (UN General Assembly, 1993) and research methodology (Straus, 1979; Dobash *et al*., 1992) has developed a very broad definition. The UN General Assembly (1993), in its Declaration on the Elimination of Violence against Women, defined violence against women thus:

‘For the purposes of this Declaration, the term “violence against women” means any act of gender-based violence that results in, or is likely to result in, physical, sexual or psychological harm or suffering to women, including threats of such acts, coercion or arbitrary deprivation of liberty, whether occurring in public or in private life. Violence against women shall be understood to encompass, but not be limited to, the following: (a) Physical, sexual and psychological violence occurring in the family, including battering, sexual abuse of female children in the household, dowry-related violence, marital rape, female genital mutilation and other traditional practices harmful to women, non-spousal violence and violence related to exploitation; (b) physical, sexual and psychological violence occurring within the general community, including rape, sexual abuse, sexual harassment and intimidation at work, in educational institutions and elsewhere, trafficking in women and forced prostitution; (c) Physical, sexual and psychological violence perpetrated or condoned by the State, wherever it occurs.’

The definition of domestic violence used by the UK government (Home Office, 2013) and its approach to eliminating violence against women and girls (UK Government, 2013) reflects the broad UN definition of gender-based violence against women. There has been discussion in the gender field as to the merits and consequences of restricting the definition of violence to physical actions or extending it to non-physical forms of abuse (DeKeseredy, 2000; Gordon, 2000; Kilpatrick, 2004; Saltzman, 2004). On the one hand are those that argue for a broad definition that includes acts of power that are not crimes so as to build concepts of a ‘continuum’ of violence (Kelly, 1988) and ‘coercive control’ (Stark, 2007). On the other are those that note that the consequences of breadth are not always desirable since they draw minor acts into the frame of violence that can obscure the extent of gender inequality found in the more severe acts (Radford, 2003). For example, Steffensmeier *et al*. (2006) find that, between 1980 and 2003 in the US, widening the definition of violent crime so as to include borderline incidents changes the gender composition of perpetrators since these borderline incidents are disproportionately perpetrated by women, thereby leading the official record to show a narrowing of the gender gap in the perpetration of violent crime. A compromise is to collect data on a wide range of forms of abuse that enables definitions data to be drawn at different thresholds to meet different definitions (Saltzman *et al*., 1999; Walby and Allen, 2004).

The third divergence concerns the importance or otherwise of the relationship between offender and victim. Recorded crime statistics do not treat this relationship as relevant so it is not recorded; but documenting the presence or absence of a domestic relationship is essential to the field of domestic violence. In mainstream crime analysis this is not usually regarded as important, so this information is rarely included in recorded crime statistics. In the gender field the relationship between offender and victim is very important; indeed in the field of domestic violence it is part of the definition, so this information is essential. The near-invisibility of the gendered relationship between offender and victim in police recorded crime statistics, since the relationship between offender and victim is not recorded there, is thus a problem for the gender field. It is certainly possible for the police to collect and record data on the relationship of the perpetrator to the victim. Indeed, there are a few minor examples of such subdivision: the categories of rape and sexual assault are subdivided by whether the victim is female or male; and there is a special category of assault for those instances where it is racially or religiously aggravated. However, in order to achieve a gender division within categories of violence against the person, it would be necessary for statute law to require this. In some countries, for example, Sweden, there is a specific offence of domestic assault against women, but not the UK. The response in the UK, and some other countries, is to find other ways to record the relationship between alleged perpetrator and offender in criminal justice system data, by the use of additional ‘flags’ but this is usually less reliable than the main statistics produced by the police, since the data collection tends to be less well resourced and the process is less rigorously audited (Walby *et al*., 2010).

The fourth concerns whether the extent of violence is counted as the number of violent events or as the number of people victimized. In the mainstream, recorded crime statistics usually count the number of offences, while the domestic violence field has tended to count the number of victims. This makes it hard to make even simple calculations such as the percentage of violent crime that is gender-based since in one case the unit is the offence and in the other it is the victim. The repeated nature of domestic violence needs to be taken into account if its nature is to be described and analysed (Farrell *et al*., 1995). But there is more than one way to do this. On the one hand, there is a call for the development of the concept of ‘coercive control’ (Stark, 2007; Myhill and Dunne, 2015) thus privileging the data unit of a ‘course of coercive control’ rather than of multiple offences. On the other hand, there is a call to increase the sophistication of the counting of frequency so as to be able to investigate whether there is a distinction between types of domestic violence, as in Johnson's (1995, 2008) typology of domestic violence that varies according to the frequency and severity of the violence between ‘intimate terrorism’ with many incidents and ‘situational couple violence’ in which there are a very few instances of low levels of violence both ways between the partners; or indeed between a largely non-victimized population and a small chronic sub-population (Hope and Norris, 2013). The way forward is to gather information using both units of measurement, victims and offences, in order to overcome this polarity.

The argument here is that if gender-based violence and domestic violence are to be mainstreamed into criminology and into sociology, then there must be a unified set of categories in which violence, both gender-based and otherwise, is measured. This might be produced in one of four ways. First, the use of mainstream categories by both fields; but this omits data that enables distinctions that are essential to the domestic violence field. Second, the use of the specialized categories by the mainstream; but that neglects the legal distinctions between crimes, so omits data that enables distinctions that are essential to the mainstream. Third, complex ad hoc acts of translation between the measurement typologies used by the two fields; but while this may be expedient on occasion it is not a satisfactory basis in the long run. Fourth, the modification of the mainstream categories in the light of the requirements of the gender violence field so that they can encompass both; which is the best long-term solution, but requires some developmental work.

## Methodology

The paper investigates the consequences of mainstreaming domestic and gendered violence for social theory. It takes as its focus to address this question, the investigation of the consequences of including domestic and gendered violence within mainstream measurements of violence and of revising mainstream measurement devices to take better account of gendered concerns. It compares the pattern of violence that is made visible before and after gender mainstreaming. By investigating the extent of domestic and gendered violence as compared with other forms of violent crime, it offers a new perspective on its scale and significance. Using a variety of measurement techniques, we investigate the extent of gender-based and domestic violence relative to other forms of violence; at each step addressing the conceptual and methodological issues in varying the measurement categories. Following the detailed empirical analysis, we address the significance of the gendering of violence for criminological and social theory. We assume that the more gender violence that is made visible by changing the techniques of measurement, then the greater is the challenge to existing theory and the greater need for its revision to be in alignment with evidence about the empirical world. Our hypothesis is that there is sufficient evidence of the large-scale extent of domestic and gendered violence to require the modification of mainstream sociological and criminological theory; however, this requires modification to the traditional categories of measurement in order to make these matters visible.

There are three gendered concepts that need to be disentangled and analysed separately: domestic violence; gender-based violence; and violence against women. So far in the paper the distinction between these terms has followed usage in the specific text under discussion. From this point onwards they are distinguished, and further distinctions are made, although they remain overlapping concepts. Domestic (in England and Wales, at least) can be divided into two further categories: intimate partners, both current and former; family members other than intimate partners. Gender-based violence is violence that is directed against a person on the basis of gender (EIGE, 2014). Violence against women is defined by the UN as ‘any act of gender-based violence that results in or is likely to result in physical, sexual or mental harm or suffering to women’ (UN General Assembly, 1993). Of course, most of this violence is primarily directed from men to women; but not all of it.

The definition and conceptualization of interpersonal violence is contested. Within mainstream crime categories, the boundary can be drawn either narrowly around the category ‘violence against the person’ or more widely so as to additionally include ‘sexual offences’ and perhaps also ‘threats’. Within the gender literature, the boundary can be drawn narrowly around acts restricted to unwanted physical and sexual contact, or extended to include threats, and further to emotional and financial abuse. We deploy more than one definition of violence in order to investigate the implications of each.

There are several sources of data that inform social science analyses of violence in the UK. These include: police ‘recorded’ crime statistics; police and CPS ‘flagged’ domestic violence; the Crime Survey for England and Wales main questionnaire and self-completion module. These will be addressed in turn using the most recent data sources in England and Wales.

## Findings

### Police recorded crime

The police produce a set of statistics known in England and Wales as the ‘Recorded crime statistics’ which record criminal offences that are reported to them and which they have recorded. These statistics do not include the relationship between alleged offender and victim, so it is not possible to construct a category of domestic violence from them. Those forms of domestic violence that are sufficiently serious to cross a criminal threshold are recorded within the crime categories; but they are not separately visible. The only form of gender-based crime that is immediately visible is that of sexual offences, since this is a gender-based group of offences, and moreover, offences in which men usually attack women. The smaller number of sexual attacks on men (usually by men) is separately identified. In addition, the published statistics on homicide are published in gender disaggregated form. Table 1, which lists the main categories of crime, shows when looking through the lens of ‘recorded crime’, domestic violence is invisible and instances of other forms of gender-based violence appear tiny.

**Table 1:** Police recorded crimes, England and Wales, 2011/12

	Number of recorded crimes	Percentage of all crime
Homicide	529	0.01
*Of which: female victims*	*171*	<*0.01*
*Of which: male victims*	*358*	<*0.01*
Sexual offences^1^	52,760	1.00
*Of which: Sexual offences against women and girls*	*34,547*	*0.90*
*Of which: Sexual offences against men and boys*	*3,548*	<*0.10*
Violence against the person^2^	626,720	16.00
Other crime	3,343,675	83.00
All crimes	4,022,626	100.00

1 Note that there are additional sexual offences which are not gendered and therefore the two subcategories of sexual offences against females and against males do not sum to the total of sexual offences.

2 Police recorded crime category of violence against the person includes homicide.

*Source*: Office for National Statistics (ONS, 2013a) 02. Appendix Tables – Crime Survey for England and Wales Year Ending September 2013: Table A4 Police Recorded Crime by Offence: Office for National Statistics (2013b) *Focus on: Violent Crime and Sexual Offences:* Figure 2.2 Homicide Offences currently recorded by the police in England and Wales by sex of victim 1996/97 to 2012/13.

### Flagged domestic violence in the criminal justice system

In order to address this invisibility of domestic and gender-based violence in UK official statistics on recorded crime, the police and Crown Prosecution Service (CPS) have started to ‘flag’ incidents and offences that are ‘domestic’ in order to ascertain their progress through the criminal justice system. The process of flagging started in 2004 and was extended in 2009. The flags are a way in which police can track the extent to which the events to which they are called out are domestic, by flagging those incidents and offences where the perpetrator was in a domestic relationship with the victim. ‘Domestic’ includes actions from partner and former partners and also by family members. ‘Domestic’ ‘flags’ are applied to both criminal offences and to incidents that do not cross a criminal threshold. A ‘domestic incident (non-notifiable crime)’ would ‘include rowdy/inconsiderate behaviour (raised voices, heated arguments etc) occurring in domestic situations involving partners (including former partners) and/or family members’ (National Policing Improvement Agency, 2009: 56). This practice begins to make domestic violence visible in police activity, with 817,000 flagged incidents recorded in 2011/12. But, in 2014, these figures do not have the standing of national statistics, their collection is not on a mandatory statutory basis, not all police forces provide them, and significant inconsistencies remain in the data (Office for National Statistics, 2013b). In addition, every police force has developed its own method of recording, making national analysis problematic.

The Crown Prosecution Service (CPS), like the police, has recently adopted the practice of flagging cases where the alleged offender, the defendant, is in a domestic relationship with the victim. However, there is a discontinuity between the two data systems, since the police count the number of offences and the CPS count the number of defendants, which means that there are no easily available statistics on the attrition of cases of gender-based violence through the criminal justice system (CJS). It is thus necessary to engage in detailed research to ascertain the extent to which the gender-based violent crimes that are recorded by the police lead to convictions in the courts. The CPS presents data on conviction rates of 73 per cent for violence against women flagged crime (CPS, 2013a), which is lower than 86 per cent for all crimes (CPS, 2013b); but these figures only address that small part of the CJS process between prosecution and conviction in courts. When detailed, but rare and ad hoc, research projects address attrition as measured from the point of recording by the police through to conviction, estimates of CJS conviction rates for gender-based violence are much lower, ranging from 6 per cent (Lovett and Kelly, 2009) to 7–8 per cent (Walby *et al*., 2010) to 12 per cent (Feist *et al*., 2007) for rape; and 2–6 per cent for domestic violence (Hester *et al*., 2008). While the use of flags is a major innovation in data collection, there is still no routine presentation of data to make visible the processing of the crimes of domestic and gender-based violence within the CJS as a whole.

### Crime Survey for England and Wales, main questionnaire

Many people do not report crimes committed against them to the police, so they cannot be included in the ‘police recorded crime’ statistics, nor in police flagged statistics, nor in the data of the Crown Prosecution Service, since their cases have not entered into the criminal justice system. This means that a considerable amount of crime is unknown to the administrative data systems of the criminal justice systems.

In response, the UK and some other countries, such as the US, have introduced surveys of their national populations in order to produce more accurate estimates of the extent of crime and changes in it over time. In the UK, a British Crime Survey started in the early 1980s and is now an annual survey, differentiated between ‘England and Wales’, ‘Scotland’, and ‘Northern Ireland’. The Crime Survey for England and Wales (CSEW) is regarded by the Office for National Statistics (ONS) as the most reliable measure of the extent of crime in its territory. The ONS (2013b: 3) states that: ‘For the crime types and population groups it covers, the CSEW provides a better reflection of the true extent of crime and a more reliable measure of trends than police recorded crime statistics. It has a consistent methodology and is unaffected by changes in levels of reporting to the police, recording practice or police activity’ (ONS, 2013b: 3). Indeed in 2014, the UK Statistics Authority (2014[Bibr b71],[Bibr b77]) temporally deselected police recorded crime statistics from the category of ‘national statistic’ because of concerns over their quality. This leaves the CSEW as the most authoritative data on crime in the UK.

The CSEW is complicated and there is a range of options for the organization and presentation of the data. We start with an account of the data as presented in official publications, and follow this by our own analysis of the raw data, in which we offer a series of methodological revisions, to better capture the extent of domestic and gendered violence. The extent of violence against the person as reported in official CSEW publications, disaggregated into three categories by the relationship of the perpetrator to the victim. These are: (a) *domestic* (current and former wife/husband/partner/boyfriend or girlfriend; other family members including son/daughter (in law); other relative; or other household member); (b) *acquaintances* (someone known to the victim, at least by sight, including: workmates/colleagues; clients/members of the public met through work; friends/acquaintances; neighbours; youths from the local area; tradesmen/builders/contactors; and (ex) husband/wife/partner of a household member); and *strangers* (c) (someone unknown to the victim). Table 2 shows the violence estimates foe 2011/12. Domestic violence appears to be 17 per cent of violent crime.

**Table 2:** Violence against the person, by domestic, acquaintance or stranger, Crime Survey for England and Wales, 2011/12, published data

	Estimated number of offences	Percentage of violent crime	Percentage of all crime
Domestic	308,000	17	3
Acquaintance	731,000	41	8
Stranger	753,000	42	8
All violence against the person^1^,^2^	1,792,000	100	19
All offences	9,500,000		100

1 CSEW is a victimization survey and therefore VAP does not include homicide.

2 Snatch theft and robbery are not included in these figures.

*Source*: Office for National Statistics (2013a) 02. Appendix Tables – Crime Survey for England and Wales Year Ending September 2013: Table A1 Trends in CSEW Incidents of Crime from 1981 to 2012/13 with Percentage Change and Statistical Significance of Change.

We reanalyse the raw data of the CSEW (ONS, 2014) in order to bring its presentation into better alignment with contemporary conceptual and methodological developments. We make two adjustments: including sexual offences; and removing the ‘cap’ (see following paragraph). We consider that sexual offences should be treated as ‘violent crime’, even though it is not in the same legal category as ‘violence against the person’, since this is the dominant understanding in the field. Prior to 2003, the category ‘sexual offences’ contained ‘consensual’ crimes of some male homosexual practices, which made its categorization as ‘violence against the person’ inappropriate. However, the gradual changing of the law (completed by the Sexual Offences Act 2003) to decriminalize most homosexual acts means that most of the remaining offences, rape and sexual assault, are more appropriately included within the category of ‘violence’.

We remove the cap on the number of offences reported to the survey that are included in its published findings. The count of the number of incidents are currently ‘capped’ so that in a ‘series’ offence, the number of offences is limited to 5 per ‘series offence’. A series offence is one in which ‘the same thing is done under the same circumstances, probably by the same people’, so contains several instances of the same offence (ONS, 2013c: 15). Although the full reported number of instances is still available, when reporting violence, the ONS caps the number of offences at 5 on the grounds that otherwise there is a risk that a small number of respondents reporting a high number of incidents will skew the overall estimates: ‘the restriction to the first five incidents in a series has been applied since the CSEW began in order to ensure that estimates are not affected by a very small number of respondents who report an extremely high number of incidents and which are highly variable between survey years … This sort of capping is in line with other surveys of crime and other topics’ (ONS, 2013c: 15).

The process of capping survey responses has been criticized both in the UK (Farrell and Pease, 2007) and in the US (Planty and Strom, 2007). It skews the estimates in a way that understates the significance of multiple offences in a course of domestic violence (and indeed other forms of violence). Domestic violence in particular is known to be an offence that is marked by repetition (see Farrell *et al*., 1995), and so, in order to capture its nature, we consider that all the incidents that respondents report to the CSEW and are recorded by the CSEW should be included in findings using CSEW data.

Our re-estimation of the extent of violence when the cap that limits the presentation of data that has been collected has been removed is shown in Table 3. We find that ‘uncapping’ significantly increases the size of the estimates of violence against the person offences and sexual offences. Thus, for all violence against the person, the ratio of the new uncapped to the old capped estimates is 1.6. This is especially where the perpetrator is known to the victim, not only in a domestic relationship (1.7 times higher for violence against the person) but also as an acquaintance (twice as high for violence against the person). The inclusion of ‘sexual offences’ in the more general category of violence is justified on conceptual grounds, but does not make a large difference to the estimates of the amount of violence.

**Table 3:** Estimated numbers of violence against the person and sexual offences, by domestic, acquaintance or stranger, Crime Survey for England and Wales, 2011/12, revised by ‘uncapping’ offences

	Estimated no. of offences ‘capped’^1^	Estimated no. of offences ‘uncapped’	Ratio of uncapped to capped violence
Domestic	315,000	526,000	1.7
Acquaintance	777,000	1,529,000	2.0
Stranger	797,000	996,000	1.2
**All violence against the person**	**1,889,000**	**3,051,000**	**1.6**
All sexual offences	*77,000	*120,000	1.6
**Violent and sexual offences**	**1,966,000**	**3,171,000**	**1.6**

* N (number of cases) is greater than 10 but less than 50 thus caution should be exercised in considering these as national estimates.

1 Our capped estimates are slightly higher than the published capped estimates because we use an alternative methodology based on prevalence, rather than incident weights. This is necessary to estimate uncapped offences.

*Source*: ONS (2014): Crime Survey for England and Wales 2011/12 accessed via the UK Data Service.

We next disaggregate these categories by gender, as shown in Tables4 and 5. Table 4 shows the gender distribution for each type of perpetrator-victim relationship, (row percentages) whereas Table 5 shows the distribution within each gender for the individual sub-types of relationship making up the three broad categories of “domestic”, “acquaintance” and “stranger” (column percentages). The gender disaggregation of the category of ‘domestic’, which has been a source of dispute in the field, shows the expected gender asymmetry of victims. Most (71 per cent) of the incidents of domestic violence that are severe enough to cross the criminal threshold are perpetrated against women, but a significant minority (29 per cent) are perpetrated against men. While (current and former) intimate partners are, as expected, the largest category of domestic violence perpetrators, that perpetrated by other family members is significant, especially against women: 38 per cent of domestic violence against women is from family members other than the intimate partner; and 28 per cent for men.

**Table 4:** Estimated number of violence offences (including sexual offences) disaggregated by gender and relationship

	FEMALES		MALES		ALL		Gender ratio (F/M)
	Estimated No.	%	Estimated No.	%	Estimated No.	%	
Domestic	419,000	71.1	170,000	28.9	589,000	100	2.46
Acquaintance	760,000	48.7	801,000	51.3	1,561,000	100	0.94
Total ‘known to victim’	1,179,000	54.8	971,000	45.2	2,150,000	100	1.21
Stranger	238,000	23.3	782,000	76.7	1,021,000	100	0.30
**Total**	**1,417,000**	**44.7**	**1,753,000**	**55.3**	**3,171,000**	**100**	**0.81**

*Note*: Figures are uncapped estimates from the Crime Survey of England and Wales 2011/12 main survey victim forms. The combined number of VAP and sexual offences may differ slightly to the total number of VAP and sexual offences given in Table 3; this is because the methodology estimates the combined total.

*Source*: ONS (2014).

**Table 5:** Estimated number of violence offences (including sexual offences) in England and Wales 2011/12 by perpetrator relationship and gender

Perpetrator	FEMALES		MALES		ALL	
	Est. No. (000s)	%	Est. No. (000s)	%	Est. No. (000s)	%
Domestic relationship						
Intimate partners including (ex)husband/(ex)wife/partner/boyfriend/girlfriend	259	61.8	*122	71.8	381	64.7
Family/household members including son or daughter (in law); other relative; other household member	160	38.2	*48	28.2	208	35.3
**TOTAL DOMESTIC VIOLENCE**	**419**	**100.0**	**170**	**100.0**	**589**	**100.0**
Acquaintance relationship						
Workmate/colleague	*38	5.0	*84	10.5	*122	7.9
Client/member of public contacted through work	265	34.8	*133	16.6	398	25.4
Friend/acquaintance	*59	7.9	124	15.5	183	11.8
Neighbour	*61	8.0	*49	6.1	110	7.0
Young people in local area	*224	29.4	172	21.3	396	25.3
(Ex) husband/wife/partner of someone in the household	*33	4.3	*6	1.0	*39	2.5
Other acquaintance	*80	10.6	*233	29.0	*313	20.1
**TOTAL ACQUAINTANCE VIOLENCE**	**760**	**100.0**	**801**	**100.0**	**1,561**	**100.0**
Stranger relationship						
Stranger	238	100.0	782	100.0	1,021	100.0
**TOTAL STRANGER VIOLENCE**	**238**	**100.0**	**782**	**100.0**	**1,021**	**100.0**

* N (number of cases) is greater than 10 but less than 50 thus caution should be exercised in considering these national estimates.

*Note*: All violence figures include sexual violence.

*Source*: ONS (2014): Crime Survey of England and Wales 2011/12 main survey victim forms.

The majority (77 per cent) of violence perpetrated by strangers is against men (23 per cent against women), as expected. But, while the amount of violence by strangers is somewhat larger (1,021,000 offences) than that by domestic perpetrators (589,000 offences), it is perhaps surprisingly less than double the amount.

Violence by acquaintances is more frequent (1,561,000 offences) than either domestic violence (589,000 offences) or by strangers (1,021,000 offences). Violence by acquaintances is very nearly as likely against women (49 per cent) as it is against men (51 per cent). Within the category of acquaintances, women are especially vulnerable to violence from a client or member of the public contacted through work (265,000 offences against women).

In total, there are nearly as many violent offences against women (1,417,000) as there are against men (1,753,000); that is, women were the victims in 45 per cent of violent offences and men in 55 per cent. This challenges the assumption that ‘violence’ is primarily a matter of men being violent to other men. The challenge to the orthodox view is only partially due to the significance of domestic violence, important as that is; it is also because of the scale of violence by acquaintances in which women are almost as frequently victimized as men.

From the perspective of violence against women, the majority (54 per cent) of the violence experienced by women is from acquaintances; this is more than from those with whom she has current or former domestic relations (30 per cent) and more than from strangers (17 per cent). This is a challenge to the orthodox view in the ‘violence against women’ field, which assumes that the majority of violence against women is from current or former intimate partners.

### Crime Survey for England and Wales, special module on intimate violence

The CSEW has two sets of questions on intimate violence: the main questionnaire where questions are asked face-to-face and entered by the interviewer in a laptop computer; and a self-completion module in which respondents enter their answers to a slightly different set of questions directly into the computer themselves thereby ensuring much greater confidentiality (Walby and Allen, 2004).

The self-completion modality of delivery of the questionnaire produces greater disclosure from victim-survivors of violence. As shown in Table 6, the self-completion module generates a 3.8 times higher rate of disclosure among victims of domestic violence than in the face-to-face questionnaire. Further, among those that answer the question on frequency of domestic abuse, the average number of incidents per victim is 7.7, which is 1.75 times higher than the face to face^1^.

**Table 6:** Crime Survey for England and Wales 2011/12, comparing face-to-face and self-completion estimates of victims of domestic violence

	Face-to-face (F2F) main questionnaire			Self-complete (SC) module		
	DOMESTIC VIOLENCE					
	Females	Males	All	Females	Males	All
VICTIMS: domestic^1^ violence^2^	133,000	*52,000	185,000	458,000	244,000	702,000
Ratio: F2F to SC				3.4	4.7	3.8

* N (number of cases) is greater than 10 but less than 50 thus caution should be exercised in considering these as national estimates.

1 Domestic includes partners/ex-partners and other family members.

2 The SC uses the CT scale rather than criminal offence categories. VAP from the SC is constructed using the CT scale items: pushed, held down, or slapped; kicked, bit, hit with fist or had something thrown at; choked or tried to strangle; used weapon; used other force; plus sexual assault.

*Source:* Crime Survey for England and Wales dataset 2011/12.

The self-completion module uses a different typology of domestic violence from the face-to-face questionnaire. This is a revised version of the conflict tactics (CT) scale that is widely used in the field (Walby and Myhill, 2001; Johnson, 1996). In addition, the module collects information on injuries from these acts in a graded typology. The CT scale is distinctive to the field of domestic violence, which is different from the conventional crime classifications. However, while the mainstream and specialized typologies appear initially to be incomparable, it is possible to make an approximate translation between the acts of the CT scale and the mainstream crime codes; though an alternative route of translation can be achieved using the injury typology (Walby, 2004). Currently, the main problem in comparing the findings of the self-completion module with the main questionnaire is lack of robust frequency data in the SC, which in its recent iterations has too limited a set of questions to enable this to be captured with accuracy.

While the limitations to the comparability of the self-completion module to the main questionnaire mean that estimates of the increased proportion of violence that is domestic, against women or sexual must be made with caution, it is reasonable to conclude that this proportion is significantly larger. Nearly four times as many people disclose experience of domestic violence in the last year in the self-completion as compared with the face-to-face questionnaire. The number of incidents that each victim discloses is also much higher in the self-completion than in the face-to-face questionnaire.

## Discussion and conclusions

Mainstreaming domestic and gender-based violence into sociology and criminology makes a difference to social theory. In order to achieve this, it is necessary to overcome several major divergences in methodology between the mainstream and gender fields. These include: the definition of violence; the conceptualization and measurement of the repetition of violence; and the making visible the relationship between offender and victim. When these revisions are made, then the scale of violence that is made visible becomes larger, and its distribution is differently gendered.

Violence against women is almost invisible in police recorded crime statistics, which have traditionally been the most authoritative account of crime, since crimes, other than the sub-categories of rape and sexual assault within the Sexual Offences category and homicide, are not disaggregated by the gender of the victim. Innovation in the recording practices in the criminal justice system by the police and prosecutors has begun to address this, but these initiatives, such as ‘flagging’ are still marginal to the main statistics. Only within the new data collection exercises of the national surveys is there serious possibility of making visible domestic and gender-based violence; but even here the issues are relatively marginalized.

The first methodological difference concerns the breadth of the definition. At one end of the continuum are the crime codes, based on the development of law, which are far behind developments in other domains, and which make violence against women nearly invisible. At the opposing end of the continuum, are definitions of violence against women that extend to almost all forms of power that are detrimental to women's well-being. We argue for the more systematic development and deployment of categories at the centre of this continuum, which would facilitate more comparability. Sexual offences are crimes and should be included within the category of violent crime. Violence against women covers both criminal and non-criminal behaviour, but significant parts of it are crimes. In order to address it as a crime, we need to include similar categories as other crimes, though revised to make gender issues visible. One of the essential revisions is to include data both on the relationships between offender and victims and also on the gender of offenders and victims, since this is necessary if the category of ‘domestic’ violence is to be made visible, and indeed the further distinctions in the categories of ‘acquaintances’ and ‘strangers’. This is available in the CSEW, but in police recorded data only through the process of flagging, which is not yet sufficiently developed for routine use. A further essential revision is to deploy mechanisms that allow for the counting of crimes that are repeats. This is especially important in the case of domestic violence, which is often a repeat offence. The survey is potentially a productive way of measuring repeated incidents, but this requires the removal of the ‘caps’ that artificially restrict the analysis of incidents that some report to surveys. These should not be treated as outliers that may be appropriately discarded, but rather an important part of the field. By defining and measuring ‘violence’ as uncapped offences of violence against the person and sexual offences, we find that the number of violent offences captured by the CSEW increases by 60 per cent compared to the published ‘capped’ count.

The violence that is made visible by delving into the raw data from the CSEW has implications for theory. We find that violence against women is not a small specialized form of practice, but a significant minority of the violence: 45 per cent of violent offences are committed against women. This is even when the full extent of domestic violence is hard to estimate accurately because of the limited data on frequency in the self-completion module of the CSEW.

The picture of violent crime as primarily constituted by men being violent to other men is wrong. There is almost as much violence against women as there is against men. It is not the case that violence is overwhelmingly from men to men.

We find that violence from strangers is a smaller proportion of violent crime than often assumed – less than a third (32 per cent). The majority of violent crime is perpetrated by someone known to the victim: an intimate partner, other family member, or an acquaintance. This is not only true for women, but also for men. It is not the case that most violence is from male stranger to male stranger: just a quarter of violent offences are committed by strangers against male victims.

Violence against women is not confined to violence from current or former partners, but is also from other family members and also from acquaintances. Violence to women is overwhelmingly from people they know at least slightly (83 per cent of violent offences). The category of ‘acquaintance’ is underestimated in current theory, which tends to polarize between, on the one hand, conceiving ‘gender-based violence against women’ as perpetrated by domestic intimates, and on the other hand conceiving of ‘violence’ as perpetrated by men to other men in public spaces.

The concept of violent crime is differently gendered than usually imagined. Mainstreaming ‘domestic violence’ into ‘violent crime’ changes the nature of the category of ‘violent crime’. Violent crime ceases to be something that primarily concerns what men do to other men who are strangers. Rather, violent crime is gendered and concerns those who are known to each other either through domestic relations or as acquaintances. This challenges the theoretical assumptions behind leading theories of crime. These are not crimes perpetrated by those who are only disadvantaged. The intersectionality of class and gender is central to the understanding of violent crime.

Hence we argue, contra Bourdieu, that it is important to acknowledge the specificity of physical violence and not to conflate it with other forms of power. And to reject his assumption of complicity, that bodies are so habituated to hierarchy that they oblige power without resistance. Further, we argue contra Žižek, that the analysis of violence is not a distraction from the analysis of more important matters. Violence in society is ubiquitous, not aberrant, with over three million physical attacks in England and Wales over the past year. As part of that, violence from people known to the victim is an important part of the structuring of the lives of many people. And, we argue, against both, that violence cannot be understood outside of its intricate gendering. These are our conclusions for sociological theory.

Our conclusion for the violence against women field starts from the finding that violence against women is more often perpetrated by acquaintances than intimates. This invites a broadening of the relevant domains so as not only to include domestic relations, but a wider range of gendered contexts. Our conclusion for criminology is that gender is more important in the patterning of violence and its theorization than currently occurs. Criminological theory should more systematically address the gendered patterns of violence in which violence against women is nearly as common as violence against men. The gendering of violence is not a marginal special issue, but should be central to the field.

## Note

Frequency of domestic abuse cannot be estimated using 2011/12 data so 2008/9 CSEW data is used for this analysis.
